# Modern Sociology

**Published:** 1899-04-29

**Authors:** 


### Modern Sociology.

### THE VISITATION OF ANGLICAN CONVENTS.

By a Correspondent.

Many touching things have been written about the blessedness of the "hidden life"—that life of consecration which is led under religious rule and in obedience to superiors and directors. "Valde magnum est in obedientia stare, sub prælato vivere, et sui juris non esse."

From Plato to the present day the realities of family life versus the theoretical perfection of community life have occupied the thoughts of all true and enthusiastic idealists. We have Shakespeare's graceful skit on academies in "Love's Labour Lost," and perhaps his dainty ridicule may still express the opinion of men of the world on schemes of supernatural perfection:—

"Light seeking light doth light of light beguile :
So, ere you find where light in darkness lies,
Your light grows dark by losing of your eyes."

We neither need nor appreciate angels; we want good men and women.

The working of the statutes of mortmain, &c., which protect the State from monks and nuns, who are held by virtue of their vows to be dead to the world and politically non-existent as individuals, is beside our present subject. We wish to start from the opposite standpoint, and to inquire what protection against possible physical or intellectual tyranny the State affords to such of her members as enter what is is called the "religious state."

The old Protestant myths about the wickedness of persecutors have few modern supporters. We have come to realise the saintly character of many eminent inquisitors. We have also come to see that every religious system has had its Inquisition—that those who undertake to "direct souls in the way of perfection" are ever prone to fanaticism. It is also self-evident that those enthusiasts who, having professed a system and rejected it, are regarded by their authorities as "relapsed," and as such are treated with a severity which is not less cruel for being fatherly.

Indifference is the price we pay for tolerance. Those who renounce the world renounce worldly indifference, and are, therefore, less tolerant than the worldling. These are mere truisms. To be practical,—How is the ordinary father to know that his daughter, professed in some Anglican convent, is not falling a victim to the misplaced enthusiasm of good but fanatical superiors? We all know that women may be led to say and do almost anything by personal influence, and the moral hold of a religious Superior over his religious subjects is hardly realisable by outsiders. Then, again, consider the power of the Corporate Spirit. The bias of a whole community is at once set against any of its members who may dare to discuss its central authority.

The moment we acknowledge that the soul is of more importance than the body we have that "little rift," which divides the body against itself. The whole being, moral, physical, and intellectual, acts and interacts, and it is the misfortune of systems to aim more or less exclusively at the discipline of one or two only of the total factors, thereby producing an abnormal and non-natural development. These facts are hardly glanced at by the young aspirant. The character of some hero stands out before the disciple as a model to be copied. No pain can be too great, no discipline too severe with such an aim before one. But the virtue to be sought is not that of some hero, but that which is "our own," την ἀριστην ἀρετην, and it is false humility to hold that our own being is less intrinsically good than the hero's. This worked out in practice means simply that if intellectual life is not killed by monastic discipline and routine, the devotée will experience a reaction, and doubts of the sanity of striving to mould the self on the plan of another person's being may lead to a state of rebellion against the authorities of a convent. Hence arises tragedy, ending either in moral suicide or rejection of the system.

Having glanced at these truths let us turn to facts. Young folk enter institutions and take vows which are recognised by their relatives. Their Superiors undertake grave responsibilities with regard to them. Henceforth they live in a seclusion into which their friends have no right to penetrate. What guarantee is there that these authorities fulfil their responsibilities?

If we read any account of a Sisterhood we see that there is always a "Visitor." This official is supposed to be responsible for the good order of the houses he visits. It is said, however, to be the custom of some "Visitors" to notify their visits in advance to the various Superiors, thus giving them time to "put their house in order" to prepare for his inspection. Obviously, such an inspection is a mere farce. In the case of episcopal visitors it is evidently somewhat difficult to arrive unexpectedly; in any case, the office is a thankless one. To announce a visitation is to prevent possibility of scandals coming to light; it is also to deprive the members of a community-house of any possible protection against fanaticism. To a "Visitor" of altruistic views complaints can be of little avail, even if they reach him; while a strong individualist may unearth cases of tyranny which would tend to lower the popularity of the order, and so lead to his own removal.

In all Sisterhoods there is a strong spirit of rivalry, and we would propose to utilise it for their mutual good. We would ask rival Superioresses to inspect each other's convents. This would give a keenness to the investigations which "Seculars" can hardly appreciate. A Sister could arrive suddenly, and no special preparations as in the case of a clergyman visitor would be needed for her reception; she could come or go without exciting attention at any time of the day or night. Novices could be interviewed by her at their work or in their cells, and details of domestic economy would come under the scrutiny of rival housekeeping experts. Superioresses would thus gain immensely in power and importance. Administrative ability, intellectual power, insight into character—these alone can fit for such a task. Elections to this office could be subjected to the Home Secretary, whose approval would place the Superioresses among officers of State. Care would be needed on both sides to maintain a good understanding between the representative of the State and her Conventual Women-Inspectors. This scheme would subject all conventual houses to a practical and stimulative discipline on their own lines. The Superioresses, too, would gain in ruling power, and the minute acquaintance with the working of rules other than their own must widen their outlook.

